# A model for superimposed coalbed methane, shale gas and tight sandstone reservoirs, Taiyuan Formation, Yushe-Wuxiang Block, eastern Qinshui Basin

**DOI:** 10.1038/s41598-022-15868-2

**Published:** 2022-07-06

**Authors:** Weidong Xie, Huajun Gan, Chongyu Chen, Veerle Vandeginste, Si Chen, Meng Wang, Jiyao Wang, Zhenghong Yu

**Affiliations:** 1 School of Earth Resources, China University of Geosciences, Wuhan, 430074 China; 2 Key Laboratory of Coalbed Methane Resources and Accumulation Process, Ministry of Education, China University of Mining and Technology, Xuzhou, 221008 China; 3 Jiangsu China Coal Geology Engineering Research Institute, Changzhou, 213000 China; 4 Department of Materials Engineering, Campus Bruges, KU Leuven, 8200 Bruges, Belgium

**Keywords:** Geology, Natural gas

## Abstract

Superimposed accumulation mechanism and model of vertical source rock–reservoir system of coal-measure gas is crucial to evaluate the exploration potential, and also the basis of co-exploration and co-production of coal measure gas. This work investigates the formation mechanism of various types of reservoirs (coalbed methane, shale gas, tight sandstone) in the Taiyuan Formation (Yushe-Wuxiang Block, eastern Qinshui Basin). Source rocks (coal seams and coal-measure mudstones) in the study area are characterized by type III kerogen, organic rich and over-mature, and reach a gas generation peak during the Early to Late Cretaceous. Coalbed methane mainly adsorbs on the surface of micropores, shale gas mainly occurs in micropores, macropores and micro-factures in adsorbed and free states, and tight sandstone gas mainly occurs in macropores in a free state. The combinations of successions are identified, coalbed methane, shale gas, and tight sandstone gas horizons are divided into a mudstone-sandstone reservoir (combination I), a coal-mudstone-sandstone reservoir (combination II), and a coal-mudstone reservoir (combination III). This division occurs from top to bottom in the succession and is identified on the basis of lithology, total organic carbon content (TOC) of mudstones, gas logging, superimposition relationships, and the source rock-reservoir-caprock assemblage. The strata thickness, continuity, and gas logging results of combination III comprise the most favorable conditions for fairly good development potential, followed by combination I. The development potential of combination II is poor due to the small strata thickness and poor continuity. The identification of superimposed reservoirs can provide an engineering reference for the exploration of coal-measure gas.

## Introduction

Coal-measure gas refers to a set of coalbed methane, coal-measure shale gas (includes shale gas and certain coal-derived gas, abbreviated as shale gas), and tight sandstone gas that are linked through genetic relationship and by vertical superimposition^1–3^. It is significant unconventional energy in terms of multi-strata, widely distributed, and resources^4,5^. China's coal-measure gas resources are extensively distributed in several provinces (autonomous regions) such as Shanxi, Inner Mongolia, Liaoning, Guizhou, Hebei, etc.^1,6^. Much fruitful exploration and development work has been performed over the past decade in these provinces. Coal-measure gas is characterized by a clear symbiotic accumulation. The gas generated by coal seam and organic-rich mudstone can fill pore space in adjacent mudstone or sandstone reservoirs in addition to self-accumulation^7,8^. Currently, coal-measure gas in the Shanxi-Taiyuan Formations of Carboniferous to Permian has the highest exploration record. However, the thickness of the different facies in the combined sequence is mostly small because of frequent facies changes in the marine-continental transitional environment, which hinder the independent development of coalbed methane, shale gas, and sandstone gas^9,10^. The exploration and development of coalbed methane have made the greatest progress in the Qinshui Basin, and there is a commercial development in the south part. However, the yield and economic benefits of some formations and blocks in this basin are not very good^11–13^. Coal-measure gas co-exploration and co-exploitation are deemed as an optimum development scheme under certain conditions, and the successful combined production of coalbed methane and tight sandstone gas in the Fuxin area of Liaoning Province and the eastern edge of Ordos Basin is encouraging^14–18^. As the understanding of the superimposed accumulation mechanism is still limited, the general application and practice of coal-measure gas co-exploration and co-exploitation still need to be further investigated, especially research on the superimposed accumulation mechanism and model of coal-measure gas resources.

The organic geochemical characteristics of source rocks, the spatial distribution of reservoirs, and gas occurrence state and space also influence the production of coal-measure gas^19–22^. Organic matter type, content, and thermal maturity affect the hydrocarbon generation type and potential of source rock, high quality gas reservoirs require source rocks to be rich in organic matter and at least in the stage of high mature thermal evolution^23–27^. Additionally, type-III kerogen has the characteristics of low hydrogen and high oxygen, containing polycyclic aromatic hydrocarbons and oxygenated functional groups^28,29^. Therefore, compared with types-I and II kerogens, it has a higher gas generation potential^28,29^. Gas occurrence state and reservoir space characteristics of different reservoirs affect the superimposed accumulation pattern. Micropores and mesopores provide a huge specific surface area and storage space for adsorbed gas, whereas free gas mainly occurs in macropores and micro-fractures^30,31^. Consequently, it is necessary to comprehensively evaluate the characteristics of source-reservoir-caprock combinations and clarify the accumulation mechanism of coal-measure gas.

Therefore, this investigation aims to clarify the hydrocarbon generation potential, spatial distribution, and storage capacity of coal-measure source rock–reservoir system based on the geological data of early coalfield and coalbed methane exploration and development; reveal superimposed characteristics of vertical source rock–reservoir system and the accumulation mechanism and model of coal-measure gas; divide the reservoir combinations of coal-measure gas in the Taiyuan Formation, and evaluate the development potential of each combination. Results from this work are a reference for coal-measure gas development in the Yushe-Wuxiang Block, and more generally, our study is significant for the understanding of the accumulation model and mechanism of coal-measure gas. Additionally, the division and evaluation of the reservoir combination have a specific engineering significance for the exploration and development of coal-measure gas.

## Geological background

**Figure 1:**
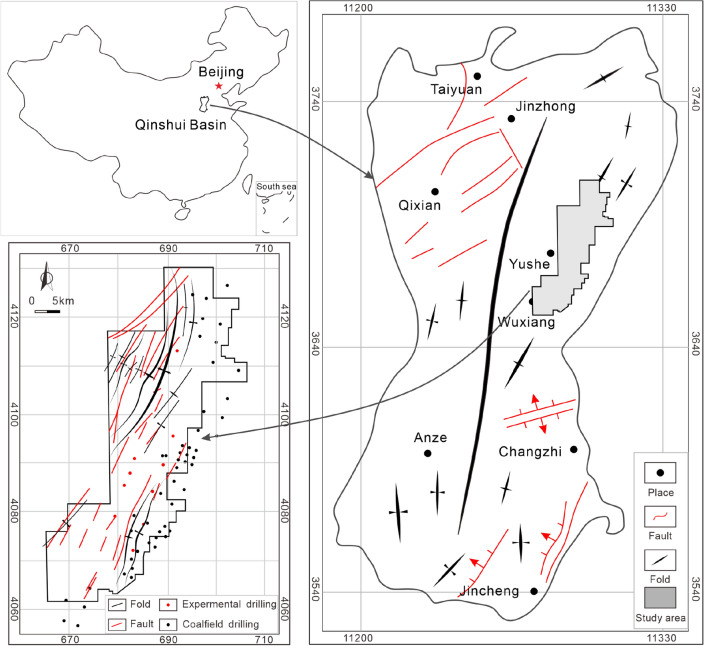
The tectonic location and structural characteristics of the Yushe-Wuxiang Block, modified from Ref.^3,53^.

Yushe-Wuxiang Block is located in the eastern Qinshui Basin and distributed in a strip shape of "wide from north to south and narrow from east to west" (Fig. 1). Qinshui Basin has suffered multi-stage tectonic movements since its formation. The transformation of the Yanshanian movement in the Cretaceous on the basin is the most significant and establishes the current tectonic framework^32,33^. A wide and gentle compound fold controls Qinshui Basin, and the strata on both sides are flat with the dip angle mostly below 5°. The structural features of faults and folds in the basin are mostly oriented along NE-SW to NNE-SSW directions controlled by the stress field of Yanshanian^34,35^. Generally, Yushe-Wuxiang Block is a monoclinal structure with NE-SW strike and NW dip (Fig. 1), and the dip angle of the strata is mostly below 10°. The exposed strata of the study area are mainly Triassic and Jurassic, and the newer strata are eroded mostly. Faults and folds are developed in the study area, and the structural features are distributed along with the NNE-SSW direction similar to the Qinshui Basin. Taiyuan Formation is deposited from the Upper Carboniferous to Lower Permian, with an average thickness of approximately 90 m. The sedimentary environment of the Taiyuan Formation is a coast-barrier island composite sedimentary system in the epicontinental sea, consisting of a tidal flat, lagoon, and barrier island on the barrier coast and a carbonate tidal flat^36^. Lithologically, it is a set of coal-measure sediments with genetic relation, comprising coal seam, organic-rich mudstone, sandstone, siltstone, limestone, etc. The bottom boundary of the Taiyuan Formation is sandstone K1 (also named Jinci sandstone), and its continuity is not very good in the study area, as it is absent in certain areas and mudstone stratum deposits. The top boundary of the Taiyuan Formation is sandstone K7 (also named Beichagou sandstone), and its continuity is also poor in the study area and it is not a good signature stratum)^13,37,38^.

Additionally, signature strata K2 (also named Maoergou limestone), K3 (also named Xiedao limestone), K4 (also named Dongdayao limestone), K5 (also named Fucheng limestone with poor stability, and usually deposit marine mudstone caused by facies changing), and K6 limestone (also named Shangou limestone) are also developed from old to young in the Taiyuan Formation^38^, among which the former three have excellent stability, while the continuity of the latter two is poor^13,37^. Coal seam 15 in the Lower Taiyuan Formation is the main coal seam and a stable signature stratum with large thickness and fairly good continuity. Moreover, it is the main production layer of coalbed methane.

## Experiment and method

TOC and vitrinite reflectance (*R*_o_) measurements are obtained using a CS230SH carbon sulfur analyzer and a DM4500P polarization microscope and QDI302 spectrophotometer. Maceral of kerogen is observed using a ZEISS Imager A2M polarization microscope. According to the standard GB/T 19,144–2010^39^, five kerogen samples with coarse size and humidity are selected. Then, the polarization microscope is adjusted to achieve 400–600 times magnification of the kerogen sample on the slide. The classification and nomination of kerogen macerals are according to the criterion of micro coal petrology (Table 1). The volume fraction of each maceral is observed by a 40 × eyepiece, and this field of view is taken as a statistical unit of exinite, vitrinite, and inertinite. Subsequently, the field of view is moved equidistantly, and more than 300 fields of view are counted for each sample. The type of kerogen is calculated by the type index of kerogen according to the standard of SY/T 5125–1996^40^ (Eq. 1). The pore volume of coal-measure gas reservoirs is full-scale characterized by high-pressure mercury (AutoPore IV 9500 mercury porosimeter) (> 50 nm), low-temperature N_2_ adsorption (1.5–50 nm) and low-temperature CO_2_ adsorption (< 1.5 nm) experiments (Quantachrome automatic specific surface area analyzer). Twelve samples (M-1–M-4, Y-1–Y-4, and S-1–S-4 are coal, mudstone, and tight sandstone samples, respectively) are conducted for the pore structure parameter tests.

1 $$ {\text{TI }} = 50*{\text{E}} - {75}*{\text{V}} - 100*{\text{I}} $$

TI is the type index of kerogen, E is exinite, V is vitrinite, and I is inertinite.

**Table 1:** Division criteria of kerogen type^36,40^.

Kerogen type	I	II1	II2	III
TI	≥ 80	40–80	0–40	< 0

Sedimentary burial, thermal evolution, and hydrocarbon generation history are reconstructed by using Petro Mod 1D and the interpolated Easy% *R*_o, max_ chemical kinetic model based on the data from Well ZK03-2 (Fig. 1). The data of top and bottom strata depth, lithology, organic geochemical parameters, etc., is obtained from drilling logging, and the erosion thickness, time, and boundary conditions refer to the results of Ref.^36^. Finally, the main input and boundary conditions information is logged and the simulation is run to acquire the simulation results.

## Generation, accumulation, and occurrence of coal-measure gas in Taiyuan Formation

### Organic geochemical characteristics and hydrocarbon generation potential of source rock

Source rock of coal-measure gas comprises coal and coal-measure mudstones (carbonaceous shale, mudstone, silty mudstone, etc.). The hydrocarbon potential is dominated by the source rock's TOC and *R*_o_. Coal (including shaly coal samples) is the accumulated organic matter with a high TOC (53.69–81.51%). Organic-rich mudstone contains dispersed organic matter, and can be further divided into carbonaceous shale (includes shaly coal and shale samples) (TOC ranges from 4.86 to 34.37%, with an average of 14.74%), mudstone (TOC ranges from 0.21 to 4.42%, with an average of 2.01%), and silty mudstone (TOC ranges from 0.28 to 9.46%, with an average of 1.98%) (Table 2). The average *R*_o_ of coal and mudstone in five drilled core samples is 2.34%, 2.87%, 2.56%, 2.67%, and 2.72%, respectively (Table 2). Organic matter is in the over-mature stage and has already passed the "liquid window", which indicates that large quantities of gaseous hydrocarbons have been generated throughout its geological history. Additionally, the kerogen type has also a specific influence on the hydrocarbon potential. The TI of kerogen in five mudstone samples is about − 78.7 to − 56.9, with an average of − 67.2 (Table 2). All TI values are lower than 0, so the kerogen samples are divided into type III, characterized by lean hydrogen and rich oxygen compared with types I and II. The parent material of type III kerogen is dominated by higher plants, which are conductive to gas generation.

**Table 2:** Organic geochemical parameters of source rock–reservoir system.

Sample ID	TI	Kerogen type	Lithology	TOC/%	Source rock-reservoir system			Accumulation type	Drilling ID	Average *R*_o_/%
					Source rock	Reservoir	Caprock			
WY5-2 (Shale)	− 56.9	III	Coal	53.69–81.51 65.52	**√**	**√**	**√**	Coalbed methane	ZK07-1	2.34
WY5-15 (Shale)	− 65.5	III	Carbonaceous shale	4.86–34.37 14.74	**√**	**√**	**√**	Shale gas	ZK03-2	2.87
WY5-31 (Shale)	− 68.4	III	Mudstone	0.21–4.42 2.01	**√**	**√**	**√**		ZK06-1	2.56
WY5-49 (Shale)	− 65.6	III	Silty mudstone	0.28–9.46 1.98	**√**	**√**	**√**		ZK08-2	2.67
WY5-73 (Shale)	− 78.7	III	Siltstone	**–**	**–**	**√**	**–**	Tight sandstone gas	ZK09-2	2.72

### Hydrocarbon generation process and period of source rock

**Figure 2:**
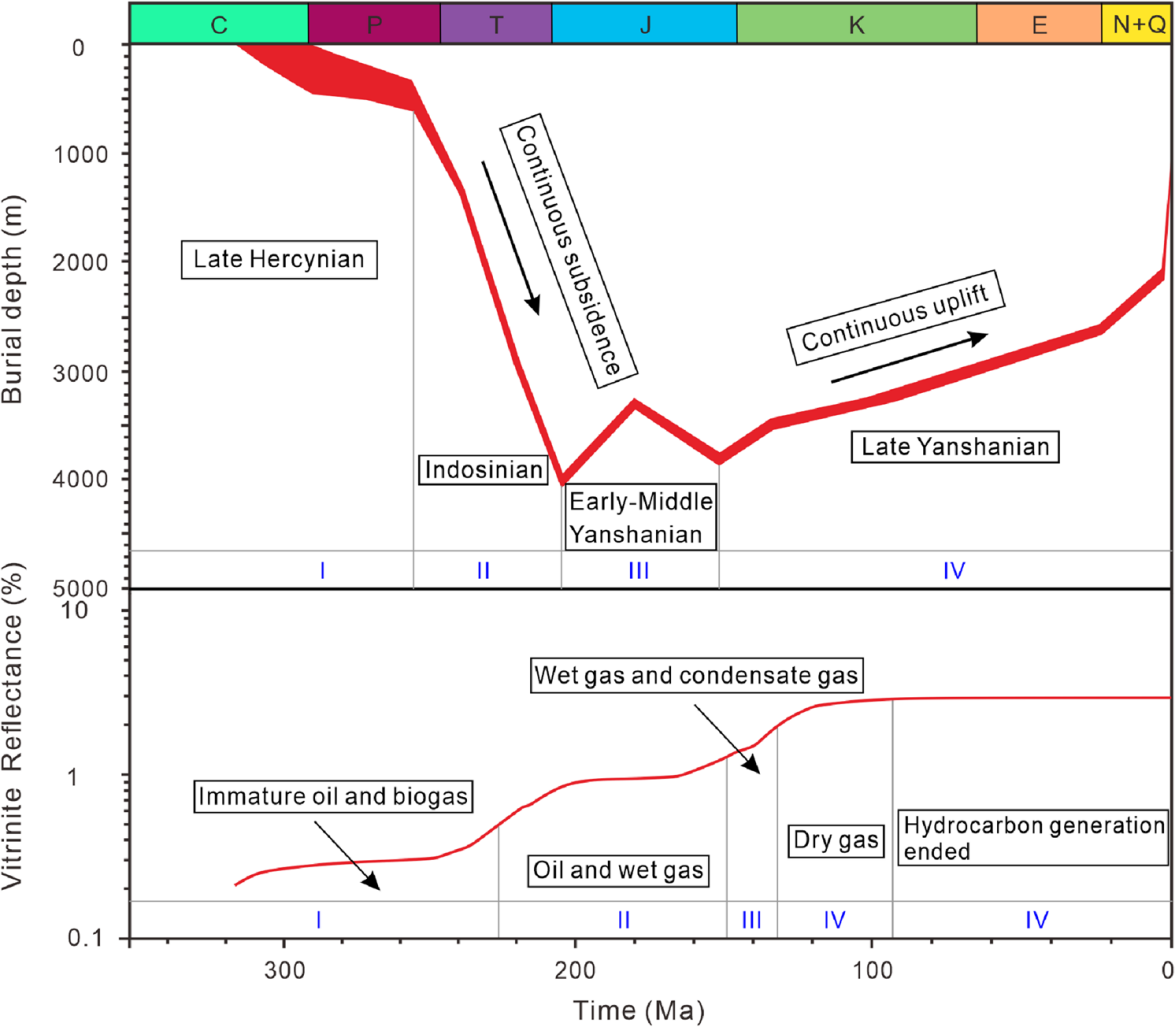
Sedimentary burial and hydrocarbon generation history of the Taiyuan Formation in the study area.

The hydrocarbon generation process and period are divided into four stages according to the sedimentary burial and hydrocarbon generation history of the Taiyuan Formation in Well ZK03-2 (Fig. 2). (1) Organic matter is immature during the Late Carbonaceous to Middle Triassic with *R*_o_ values in the range of 0–0.5%. Only a little immature oil and biogas are generated, which is promoted by biochemical action. (2) Organic matter evolves to the mature stage during the Middle Triassic to Late Jurassic with *R*_o_ values in the range of 0.5–1.3%, oil and wet gas are major products promoted by asphaltization and aromatization. (3) Organic matter evolves to the high-mature stage during the Late Jurassic to Early Cretaceous with *R*_o_ values in the range of 1.3–2.0%, wet gas and condensate gas are the main products promoted by aromatization, cyclocondensation, and pyrolysis. (4) Organic matter evolves to the over-mature stage during the Early Cretaceous to Late Cretaceous with *R*_o_ values in the range of 2.0–3.0%, dry gas generation peak is reached promoted by thermal cracking (demethylation). Then, significant hydrocarbon generation ends caused by tectonic continuous uplifting. The coal-measure gas reservoir is transformed and adjusted, resulting in the current occurrence characteristics.

### The occurrence space of coal-measure gas reservoirs

**Figure 3:**
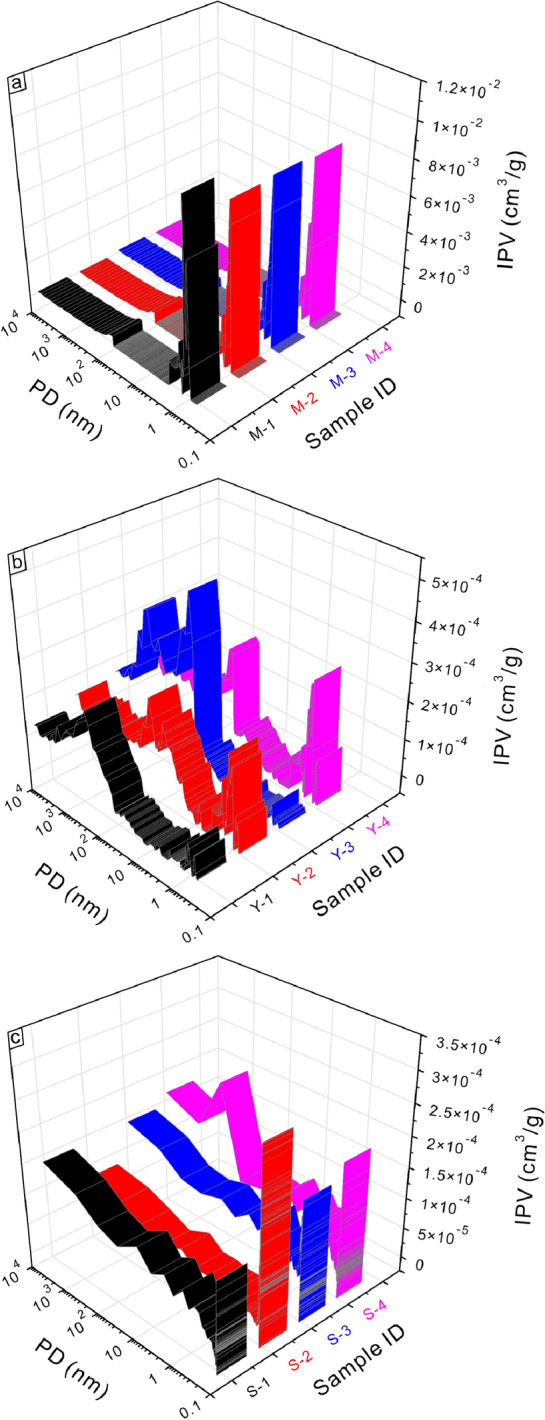
Incremental pore volume versus pore size distribution of coal-measure gas reservoirs. PD is pore diameter, IPV is incremental pore volume; (a), (b), and (c) is the incremental pore volume (0.3–10,000 nm obtained from high-pressure mercury (> 50 nm), low-temperature N_2_ adsorption (1.5–50 nm) and low-temperature CO_2_ adsorption (< 1.5 nm) experiments) of coal (M-1–M-4), mudstone (Y-1–Y-4), and sandstone (S-1–S-4) samples versus pore diameter.

Full-scale pore volume, advantage pore size interval, gas storage capacity, and pore connectivity of the coal-measure gas reservoirs are discussed (Fig. 3). Coal seams' incremental pore volume versus pore size distribution curves are characterized by "unimodal–micropores dominated" (Fig. 3a). Micropores with a pore diameter in the 0.9–2 nm range are developed best, followed by the mesopores in the 2–3.3 nm range, whereas pores with a pore diameter greater than 3.3 nm are developed poorly, which indicates that the pore connectivity of the coal reservoir is less good. Pores in mudstone reservoirs have characteristics of "multimodal–micropores and macropores dominated" (Fig. 3b). Micropores in the 0.7–2 nm range and macropores greater than 100 nm are well developed, whereas the mesopores and macropores in the 50–100 nm range are developed poorly. Pore connectivity of mudstone reservoirs is medium, slightly better than that of coal reservoirs. Pores in sandstone reservoirs are characterized by "multimodal-full-scale coexistence" (Fig. 3c), indicating that sandstone reservoirs' pore connectivity is excellent. Overall, the pore volume of coal reservoirs is most developed, which is over an order of magnitude greater than that of mudstone reservoirs, and the pore volume of sandstone is the lowest. However, the pore connectivity sequence of coal, mudstone, and sandstone reservoirs are opposite to pore volume.

## Superimposed accumulation mechanism and model of coal-measure gas

### Continuity of coal-measure gas reservoirs

**Figure 4:**
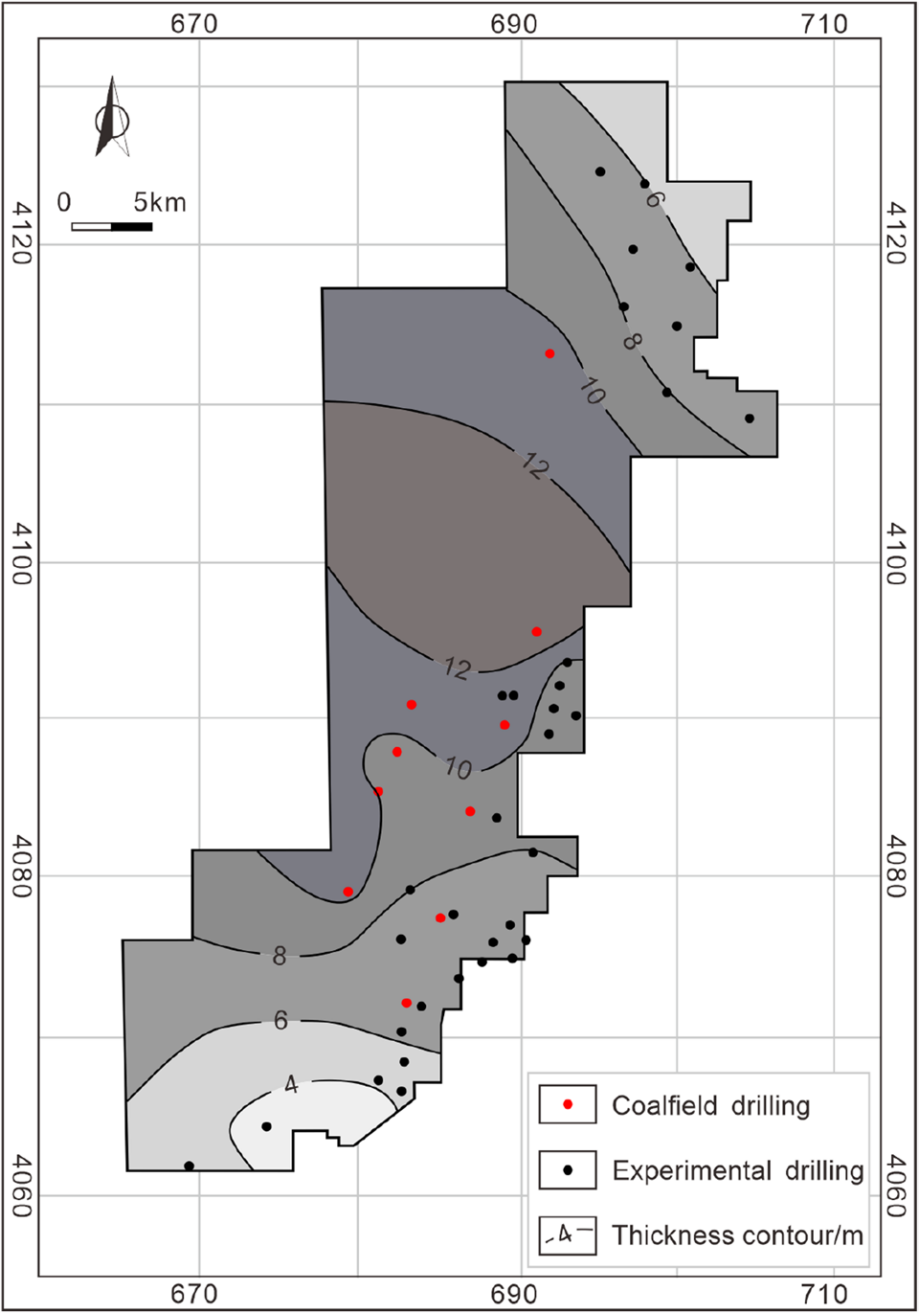
Cumulative thickness contour of coal seams in the Taiyuan Formation.

**Figure 5:**
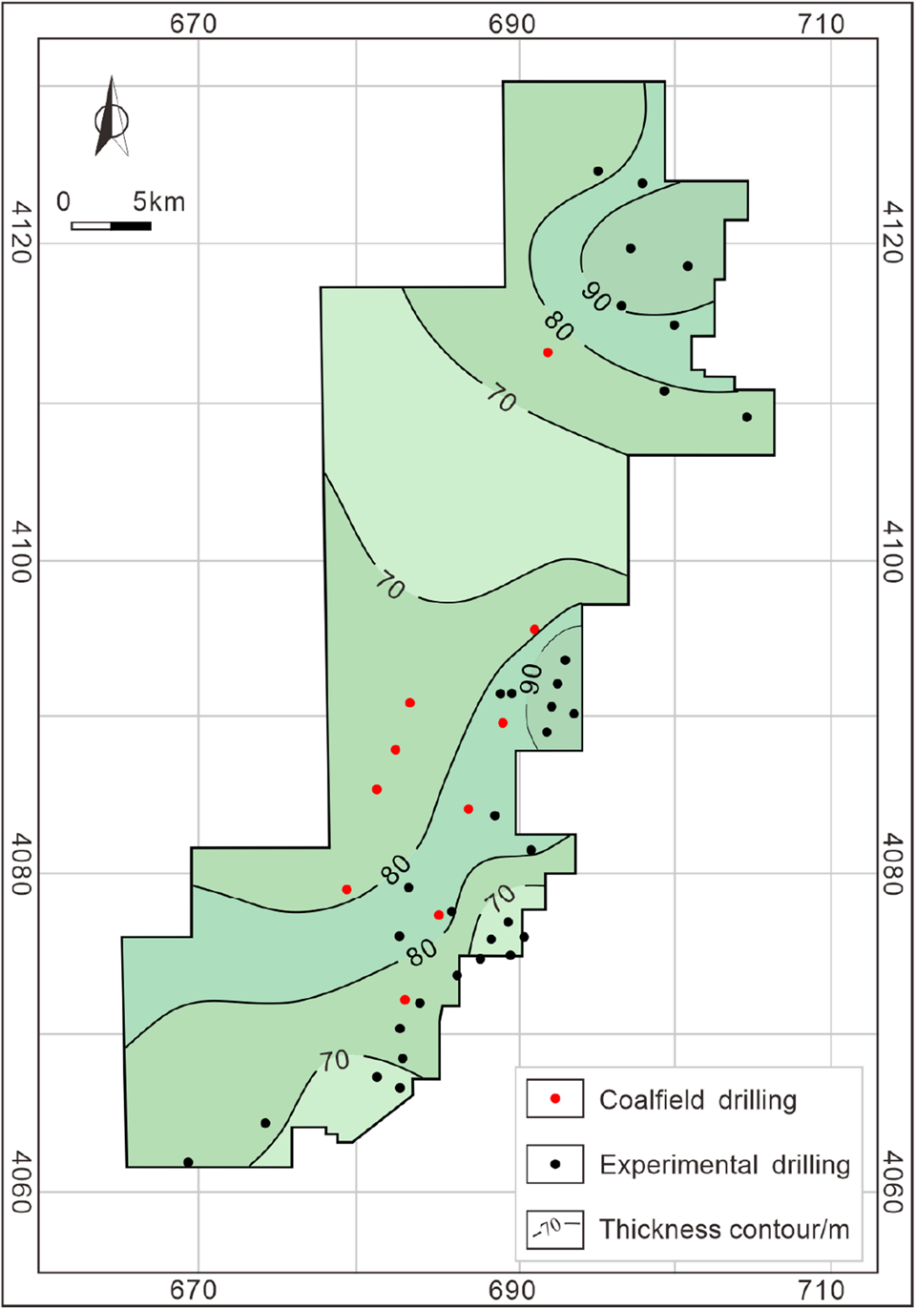
Cumulative thickness contour of mudstone in the Taiyuan Formation.

**Figure 6:**
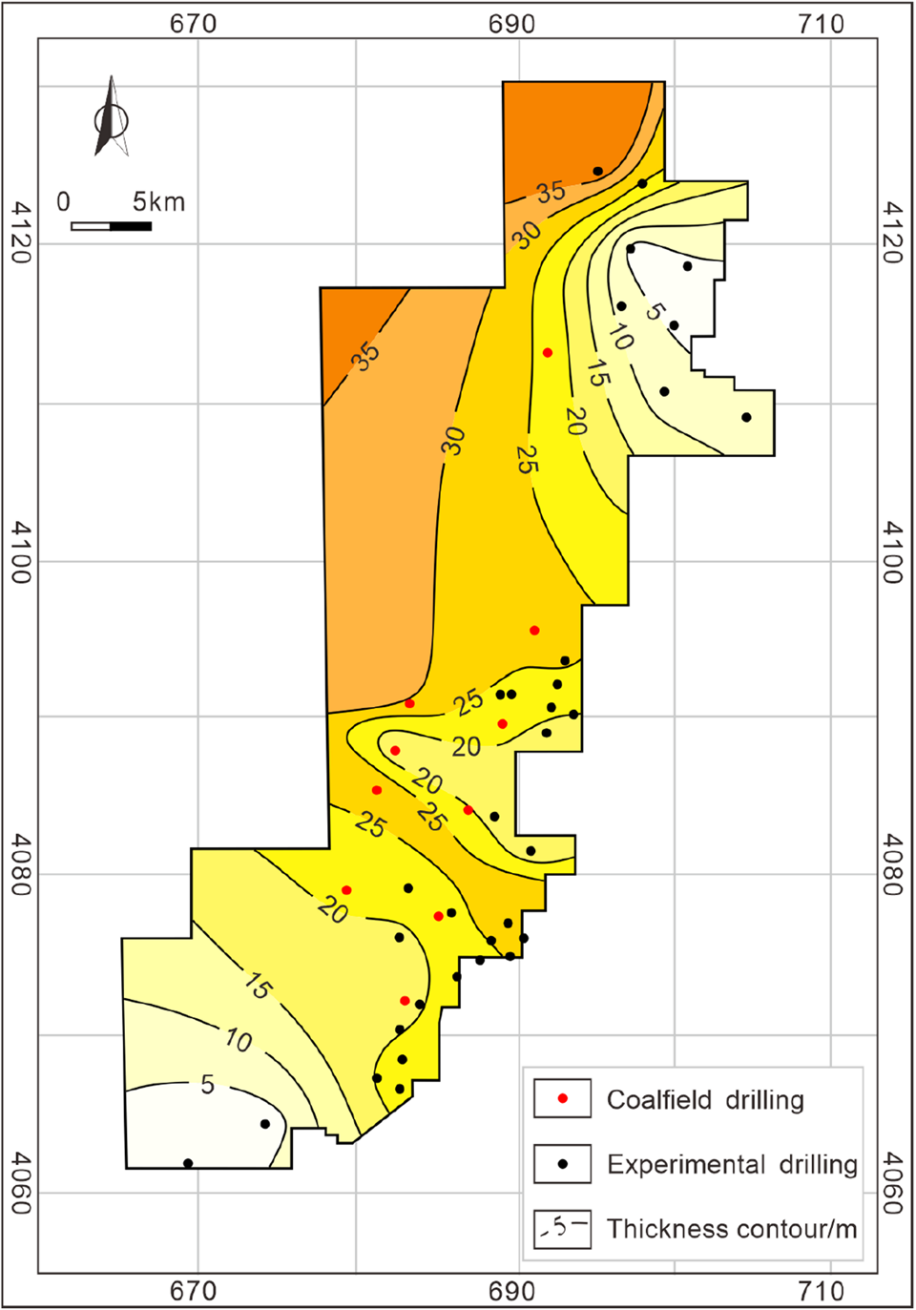
Cumulative thickness contour of sandstone in the Taiyuan Formation.

As mentioned above (Sect. 3), organic geochemical parameters confirm that the coal-measure source rock has good hydrocarbon generation potential and experiences the peak of both oil and gas generation during its geological history, laying the foundation of coal-measure gas accumulation. Coalbed methane and shale gas are characterized by self-generating and self-storing properties, whereas tight sandstone gas in coal measures is a heterogeneous gas reservoir, which needs to be filled and accumulated by the short-distance migration of gas from adjacent coal seams or mudstone. Taiyuan Formation contains 10–14 coal seams with a cumulative thickness ranging from 3.01 to 13.84 m (8.34 m averages) (Fig. 4), of which coal seam 15 in the lower part of the Taiyuan Formation is the main coal seam. The maximum cumulative thickness of coal seams (greater than 12 m) is in the middle of the study area, and the thickness gradually decreases to the north and south sides. The cumulative thickness of mudstone is large and stable, ranging from 52.92 to 110.84 m (mainly in the range of 70–90 m) (Fig. 5). However, controlled by the frequent facies changes in the sedimentary environment, the thickness of a single mudstone layer is thin, and sandstone, limestone, and coal seam interlayer are widely developed, resulting in poor continuity. The thickness stability of sandstone in the Taiyuan Formation is poor, ranging from 2.2 to 46.59 m (Fig. 6), with an average of 22.27 m. Besides, the single-layer thickness of sandstone is small, mostly thinner than 5 m. The thickness and continuity of the reservoir are the basis for the resource and development potential of shale gas and tight sandstone gas reservoir^41,42^. Therefore, the characteristics of thin single-layer thickness and poor spatial continuity of these kinds of reservoirs in the study area limit its independent development benefits, and the results of exploration and development practice have also proved this^9,10^.

**Figure 7:**
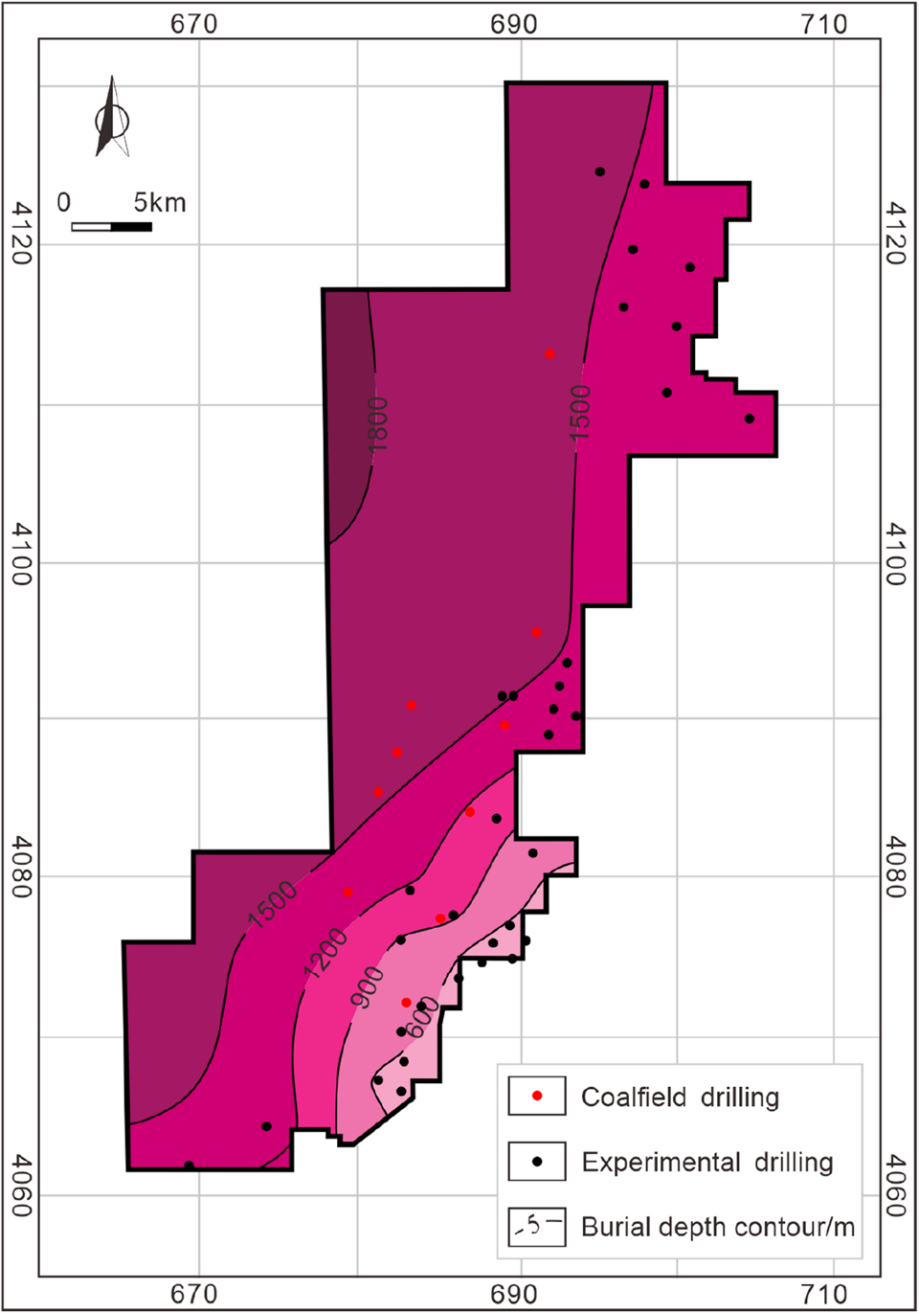
Burial depth contour of the Taiyuan Formation in the study area.

**Figure 8:**
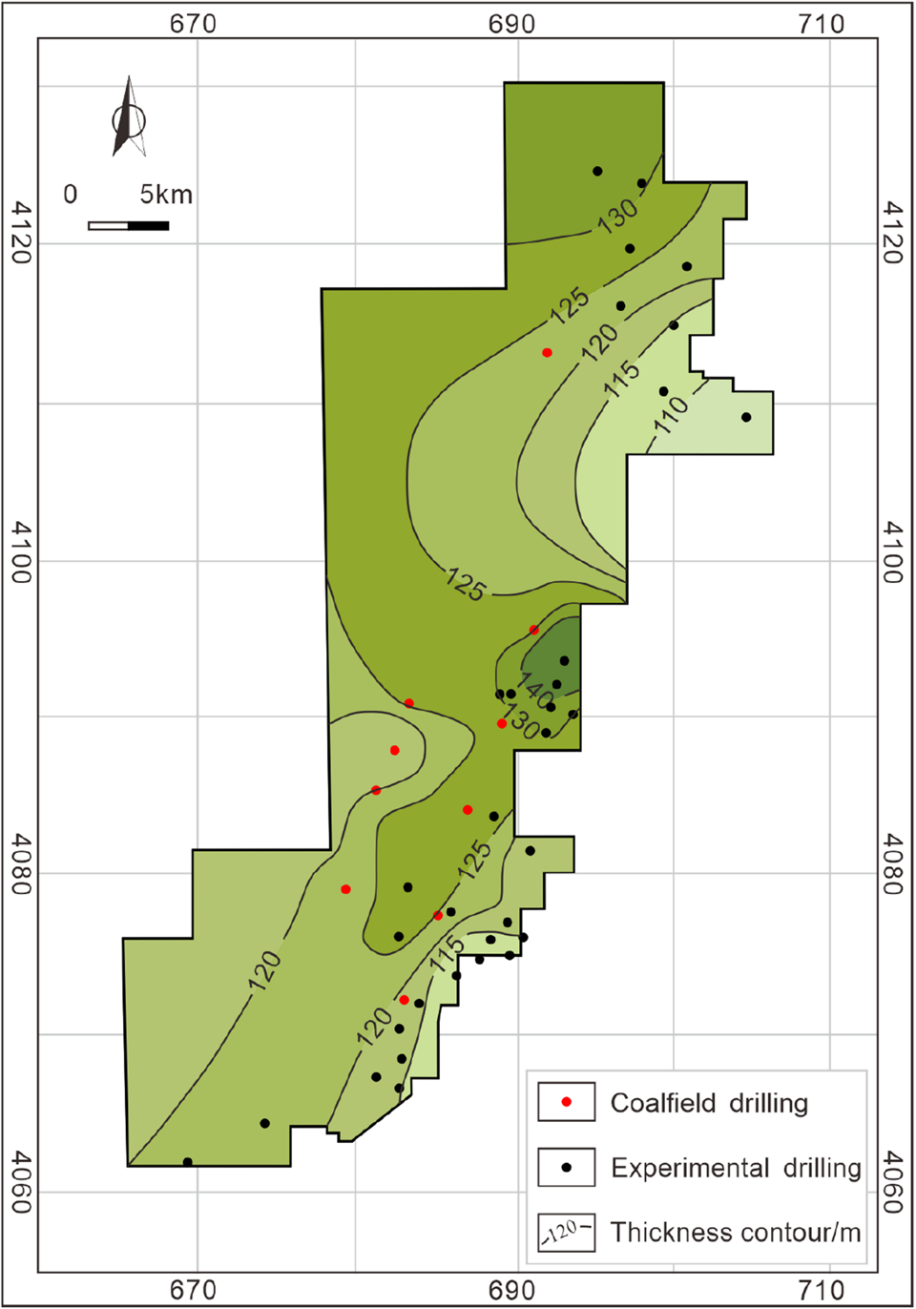
Thickness contour of the Taiyuan Formation in the study area.

The burial depth of the Taiyuan Formation is in the range of 400–1800 m, and gradually deeper along with the SE-NW direction. Except for the southeastern corner of the study area, most of the burial depths are greater than 1000 m (Fig. 7). The cumulative thickness of coal-measure gas reservoirs is large, ranging from 102 to 157 m (123 m averages). Most areas are in the range of 120–130 m with fairly good lateral stability (Fig. 8). Hence, co-exploration and co-exploitation of coalbed methane, shale gas, and tight sandstone gas could be a promising scheme^1,2^. Hence, it is necessary to clarify the vertical superimposed accumulation mechanism and model of coalbed methane, shale gas, and tight sandstone gas in coal measures.

### Superimposed accumulation mechanism and model

**Figure 9:**
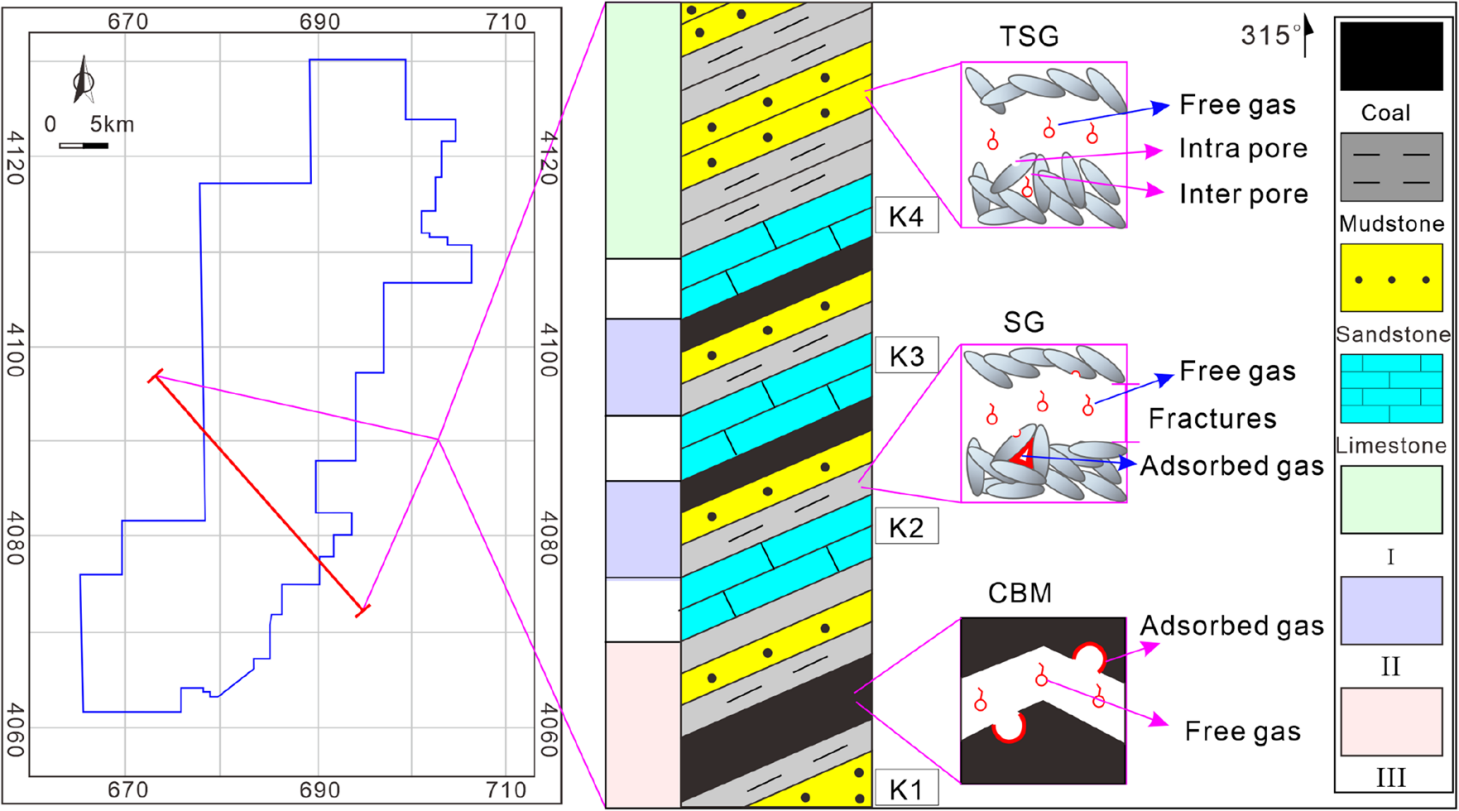
Coal-measure gas superimposed accumulation model, mechanism, and combination of the Taiyuan Formation. TSG is tight sandstone gas (mainly free gas), SG is shale gas (including free gas and adsorbed gas), CBM is coalbed methane (mainly adsorbed gas), Intra pore is the intragranular pore, and inter pore is the intergranular pore, I–III represents combinations I–III. The gas occurrence states and pore types are drawn referring to ref^43–47^.

**Figure 10:**
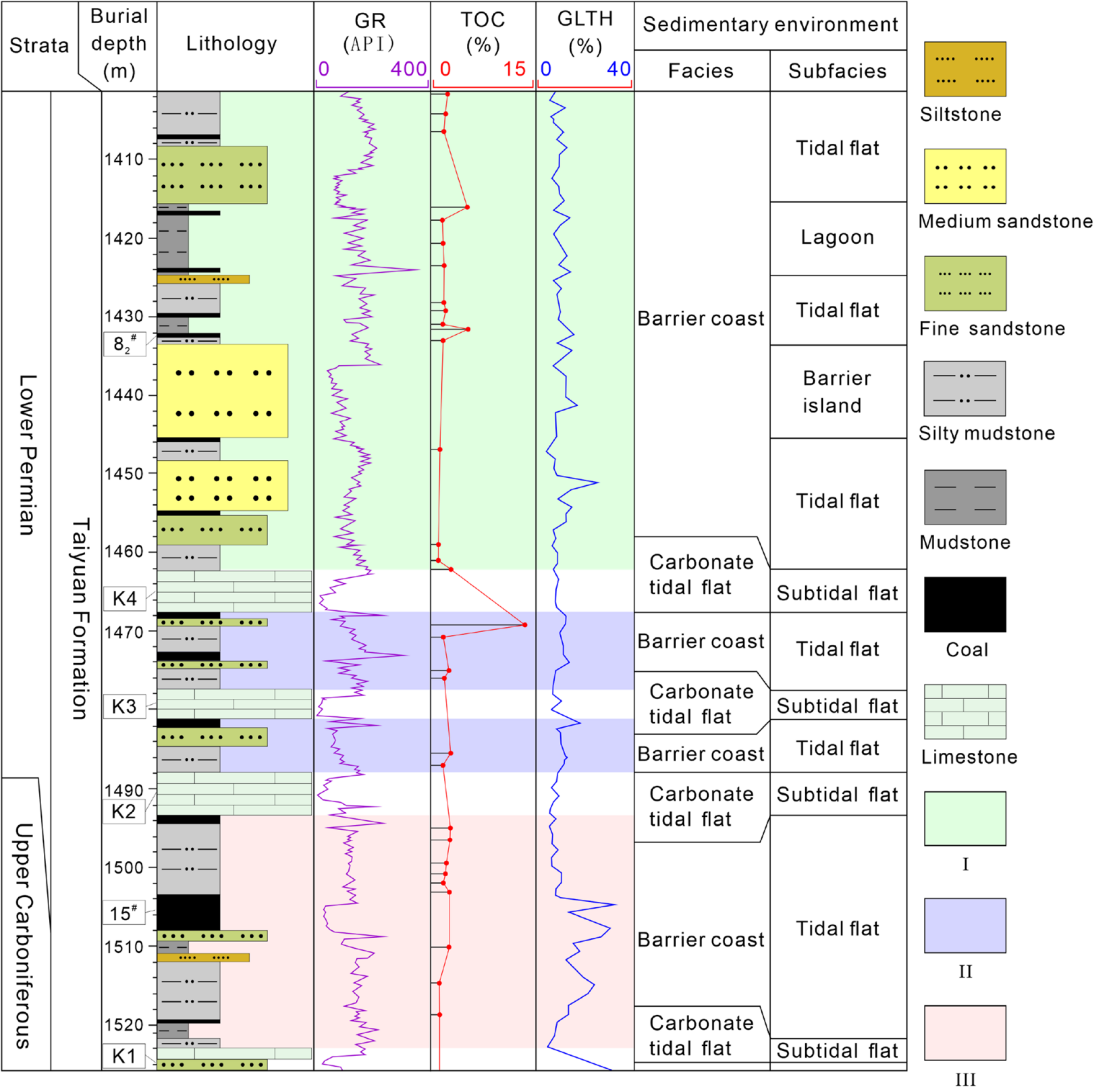
Reservoir combinations and resource potential of the Taiyuan Formation in Well ZK08-2. GR is natural gamma-ray logs, TOC is total organic carbon content, and GLTH is gas logging total hydrocarbon content. I–III represent combinations I–III.

Coal-measure gas reservoirs of the Taiyuan Formation are deposited from the Late Carboniferous to Early Permian, coalbed methane, shale gas, and tight sandstone gas are superimposed and accumulated vertically. Their occurrence mode and accumulation mechanism are distinct, resulting in different accumulation models (Fig. 9). Adsorbed gas is dominant in coalbed methane and mainly occurs in micropores and cleats. Shale gas mainly occurs in organic matter pores, intergranular pores, intragranular pores, and micro-fractures in adsorbed and free states^43–45^. Whereas free gas is dominant in tight sandstone reservoirs and mainly occurs in intragranular pores^46,47^. The accumulation process of coalbed methane and shale gas is controlled by the sedimentary burial and thermal evolution of source rocks, which have experienced multi-stage oil and gas filling and reached the gas generation peak in the Cretaceous (Fig. 2). Ju et al.^48^ and Liang et al.^49^ suggested that only about 10% of the natural gas generated from coal is stored and retained in coal seams, and the rest is lost or filled into adjacent reservoirs. Shale gas accumulation is characterized by an analogous process^50,51^. Consequently, the accumulation of tight sandstone gas is closely related to the hydrocarbon generation and expulsion of coalbed methane and shale gas reservoirs^48–51^. The supply of a tight sandstone reservoir includes two ways: (1) the source rock reaches the gas generation peak during the Cretaceous. Coal seams and shale gas reservoirs are in the ultra-high-energy gas-bearing state. The gas continues to fill and expand the reservoir, resulting in a pulsating caprock breakthrough vertically. Moreover, the gas migrates to adjacent sandstone reservoirs driven by the concentration gradient and the pressure gradient^48,49^. (2) The main hydrocarbon ends in the Late Cretaceous, the burial depth, in-situ temperature and pressure of the reservoir decreases, and the quantity of fault and fold increases due to continuous tectonic uplifting, which leads to a change of preservation conditions of the gas reservoirs. Specifically, the adsorbed gas in the coal seam and shale gas reservoirs is desorbed and transformed to a free state, and the storage capacity of the reservoir is also reduced. Hence, excess gas penetrates the reservoir and migrates to the adjacent sandstone gas reservoirs continuously until the gas storage in coal seams and shale gas reservoirs reach an equilibrium state. Li et al.^52^ also proved that tight sandstone gas in the Qinshui Basin is characterized by mixed sources based δ ^13^C isotope measurements in the saturated hydrocarbon, which are supplied by both adjacent coalbed methane and adjacent shale gas reservoirs. Finally, superimposed coal-measure gas reservoirs are formed vertically (Figs. 9 and 10).

### Reservoir combinations of coal-measure gas

**Figure 11:**
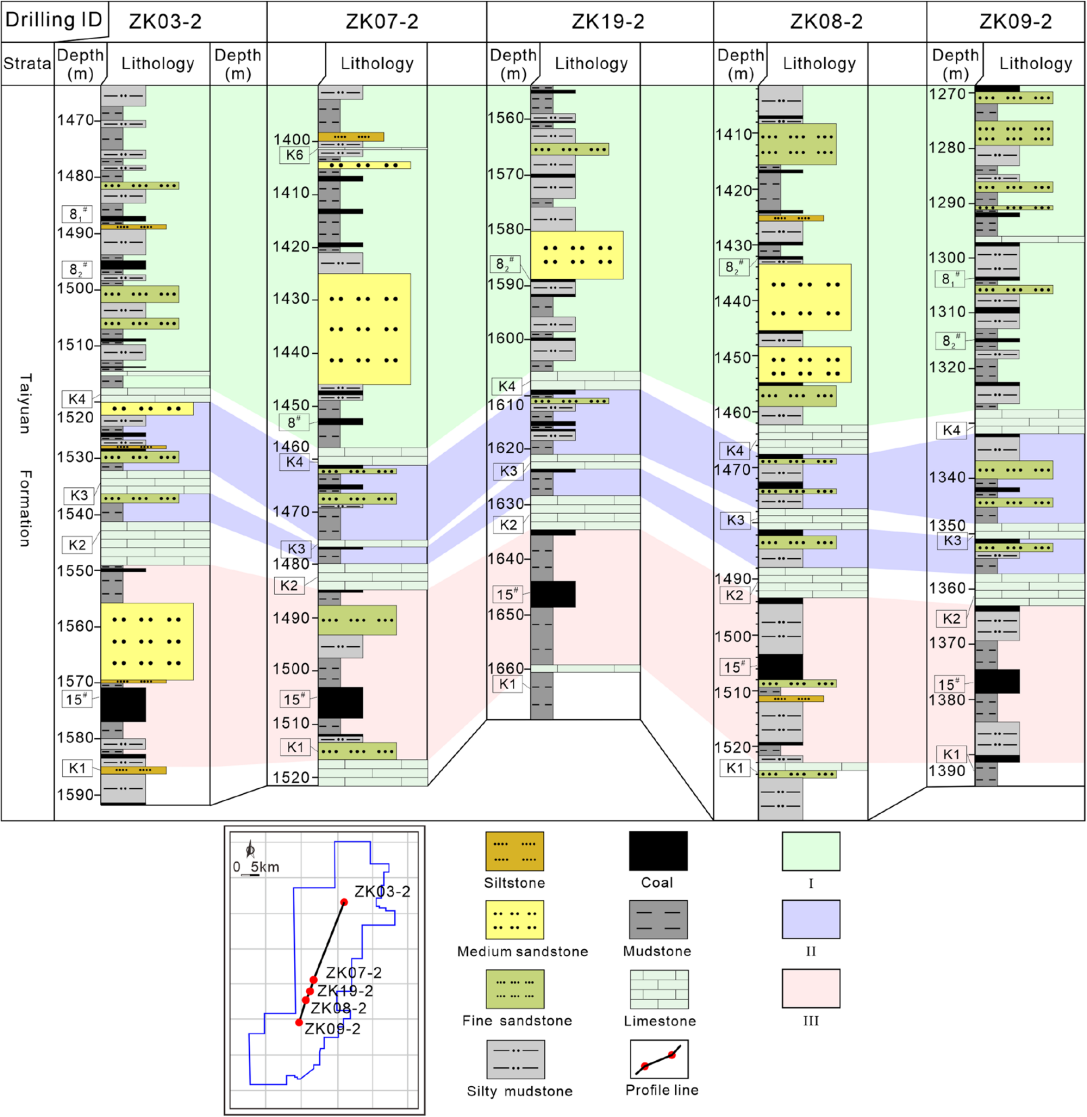
Spatial distribution characteristics of combinations I, II, and III in the study area. K1–K7 are the signature strata of Taiyuan Formation, and 8_1_^#^, 8_2_^#^, 15^#^ are coal seams. I–III represent combinations I–III.

As mentioned above, the co-exploration and co-exploitation of coalbed methane, shale gas, and tight sandstone gas are deemed as promising scheme. Accordingly, the reservoir combination of coalbed methane, shale gas, and tight sandstone gas is divided into mudstone-sandstone reservoir (combination I), coal-mudstone-sandstone reservoir (combination II), and coal-mudstone reservoir (combination III) according to the lithology, continuity, TOC of mudstone, gas logging, superimposed relationship, and source rock–reservoir–caprock assemblage (Figs. 10 and 11), and the exploitation potential of each combination is discussed correspondingly.

1. Reservoir combination of mudstone-sandstone.

The combination I is developed in the upper part of the Taiyuan Formation with limestone K4 and sandstone K7 (it is the top boundary of the Taiyuan Formation when K7 is missing) as the bottom and top boundaries, respectively (Fig. 10). The total thickness is greater than 60 m. Lithology is dominated by interbedded mudstone and sandstone, and several thin coal seams interlayers are developed. It is deposited in the tidal flat, lagoon, and barrier island of the barrier coast. Mudstone and coal seams are the source rocks with high organic matter abundance, large accumulative thickness, and sufficient gas sources. Sandstone is distributed among mudstone; coalbed methane and shale gas could fill sandstone after short-distance migration to form the tight sandstone gas reservoirs. Sandstone reservoir is distributed among mudstones with good sealing capacity. Additionally, the burial depth of combination I is moderate, ranging from 1400 to 1600 m, and the large accumulative thickness and favorable continuity are conducive to gas preservation. In general, gas logging of combination I is medium, and the sandstone section at the bottom is the best.

(2) Reservoir combination of coal-mudstone-sandstone.

The middle part of the Taiyuan Formation develops two sets of combination II, which are between limestone K2 and K3, and K3 and K4, respectively. The thickness of combination II is approximately 10 m. Lithology is dominated by sandstone, coal, and mudstone from old to new with strong rhythmicity, which is mainly deposited in the tidal flat of the barrier coast. Combination II has complete source rock-reservoir-caprock assemblage, and the gas logging is medium, in which gas logging of coal seam and its adjacent strata is better than mudstone and sandstone, and TOC distribution of mudstone also presents similar regularity. The overlying and underlying limestone of combination II has a large and stable thickness, good continuity, dense lithology, and strong sealing ability in the study area. Two sets of combination II are buried in the depth of 1466–1490 m with a moderate burial depth, which is favorable to coal-measure gas exploitation. Nevertheless, the thickness of the two sets of combination II is much less than 30 m, limiting the gas reservoirs' continuity and development potential.

(3) Reservoir combination of coal-mudstone.

The combination III is developed between sandstone K1 and limestone K2 at the lower part of the Taiyuan Formation with a thickness of about 35 m. The lithology is dominated by mudstone with an accumulative thickness greater than 20 m, followed by coal seams (mainly coal seam 15) with a thickness of about 5 m. Additionally, several thin sandstone interlayers are developed. In general, combination III exhibits fairly good gas logging, and the middle and the lower part are better than the upper part. The exploration and development of coalbed methane in coal seam 15 in the Qinshui Basin also presents a high gas content^53,54^. The burial depth of combination III is in the range of 1493–1528 m with a thickness greater than 30 m, and the preservation conditions and continuity are good. Accordingly, combination III has the greatest development potential relative to combinations I and II due to source rock–reservoir–caprock and gas logging characteristics.

### Continuity of the accumulation combinations

According to the spatial configuration of the study area, five exploratory drillings are selected from north to south, and the three reservoir combinations of the Taiyuan Formation are divided, respectively (Fig. 11). Then, the connecting-well profile of five drillings is drawn, and the three accumulation combinations' spatial continuity and exploitation potential are discussed. The thickness of combination I is in the ranges of 50–70 m with a good continuity as a whole. Its lithology is dominated by mudstone, while thick sandstone deposits in the barrier island environment within the region of Well ZK07-2, ZK19-2, and ZK08-2 in the middle of the study area. The thickness of those sandstone layers gets thinner in the region of Well ZK03-2 and ZK09-2 (northern and southern part of the study area) (Fig. 11), resulting in a worse stability. Additionally, there is a distinction in the amount and thickness of the intercalated coal seams. The burial depth ranges from 1270 to 1610 m, and the development potential is good.

Combination II between limestone K2 and K3 is thin, ranging from 4 to 6 m, and the strata in Well ZK03-2, ZK07-2, and ZK19-2 are incomplete. Coal seam or sandstone is missing. The thickness of combination II between limestone K3 and K4 is in the range of 10–16 m, and the distribution of coal seam, sandstone, and mudstone is stable. In general, the thickness of combination II is too thin, resulting in poor resources, continuity, and development potential. The thickness of combination III is large and stable, ranging from 30 to 35 m, and the continuity is good. The burial depth is between 1360 and 1670 m, source rocks are stably developed, and the sealing ability of reservoirs is favorable so that the preservation conditions and development potential of combination III are excellent and greater than that of combinations I and II. In addition, faults and folds are developed in the study area (Fig. 1). The influence of structure should be considered in the process of exploration and development. Generally, faults are not conducive to reservoir continuity and preservation conditions, especially normal faults^55,56^. The wide and gentle monocline is the best gas enrichment area, and the well-sealed anticline core is also conducive to gas accumulation^57,58^.

It should be noted that although large-scale exploration of coal-measure shale gas and tight sandstone gas has been carried out and industrial gas flow has been drilled in some areas, their commercial development is hindered by frequent transgression and regression of transitional facies, rapid facies changing of coal measures sediments and thin single-layer thickness^59^. Currently, only coalbed methane in Taiyuan formation has realized large-scale commercial development, the independent development effect of shale gas and tight sandstone gas is difficult to meet the expectation. The co-exploration and co-production of coal-measure gas is a development scheme with great potential, but its implementation is still limited by the complex reservoir accumulation mechanism and engineering difficulty, especially the exploitation pattern of coal measure gas and the geological dynamic recognition of co-production layers.

## Conclusion

Coal seam and mudstone are coal-measure gas's source rocks, characterized by type III kerogen, organic-rich, and over-mature. The gas generation peak of source rocks is reached during the Early Cretaceous, and coalbed methane and shale gas have typical characteristics of source-reservoir integration. However, the tight sandstone gas is partially supplied by adjacent coalbed methane and shale gas reservoirs, including two main processes of coalbed methane and shale gas pulsate caprock and migrate to adjacent sandstone reservoirs driven by the concentration gradient and pressure gradient during the gas generation peak; and tectonic uplifting leads to a lower storage capacity of mudstone and coal seam, and the excess gas penetrates the reservoir and migrates to sandstone reservoirs.

Pores in coal reservoirs are characterized by "unimodal–micropores dominated" and the micropores in the 0.9–2 nm range are most developed. Pores in mudstone reservoirs have the characteristics of "multimodal–micropores and macropores dominated". Micropores in the 0.7–2 nm range and macropores greater than 100 nm are well developed. Pores in sandstone reservoirs are characterized by ‘multimodal–full-scale coexistence’. The pore volume of coal reservoirs is developed largest, over an order of magnitude greater than mudstone and sandstone reservoirs. Three reservoir combinations of the Taiyuan Formation are divided for co-exploration and co-production. The exploitation potential of combination III is the best with moderate burial depth, large thickness, good continuity, and high gas logging, followed by combination I; whereas the exploitation potential of combination II is poor due to its thin thickness and poor continuity.

## Data Availability

The datasets generated and/or analyzed during the current study are not publicly available due to the requirements of the funding project but are available from the corresponding author on reasonable request.

## Abbreviations

Combination I: Mudstone-sandstone reservoir
Combination II: Coal-mudstone-sandstone reservoir
Combination III: Coal-mudstone reservoir
TOC: Total organic carbon content
*R*_o_: Vitrinite reflectance
K1: Jinci sandstone
K2: Maoergou limestone
K3: Xiedao limestone
K4: Dongdayao limestone
K5: Fucheng limestone
K6: Shangou limestone
K7: Beichagou sandstone
